# Co‐Occurring Conditions and Sleep Symptoms Associated With Obstructive Sleep Apnea in Children With Down Syndrome

**DOI:** 10.1002/ppul.71376

**Published:** 2025-11-24

**Authors:** Taylor A. Curry, Emily Cooper, John Brinton, Kristine Wolter‐Warmerdam, Norman R. Friedman, Stephen M. M. Hawkins, Francis Hickey, Benjamin H. Hughes, Emily M. DeBoer

**Affiliations:** ^1^ Department of Pediatrics Indiana University School of Medicine Indianapolis Indiana USA; ^2^ Department of Biostatistics and Informatics Colorado School of Public Health Aurora Colorado USA; ^3^ The Breathing Institute Children's Hospital Colorado Aurora Colorado USA; ^4^ Sie Center for Down Syndrome Children's Hospital Colorado Aurora Colorado USA; ^5^ Department of Otolaryngology University of Colorado School of Medicine Aurora Colorado USA; ^6^ Department of Pediatrics University of Colorado School of Medicine Aurora Colorado USA

**Keywords:** altitude, children, down syndrome, OAHI, obesity, sleep apnea

## Abstract

**Background and Objectives:**

Sleep disordered breathing (SDB) is prevalent in children with Down syndrome (DS). The American Academy of Pediatrics recommends that all children with DS undergo a polysomnogram between 3 and 4 years of age irrespective of symptoms. Our objective is to describe the clinical symptoms and breathing patterns of children with DS based on their polysomnogram results.

**Methods:**

A large, single‐center retrospective study evaluated SDB in children with DS at moderate altitude between 1642 and 2087 m above sea level. The primary outcome was obstructive apnea hypopnea index (OAHI). Secondary outcomes included central apnea index (CAI), gas exchange, sleep symptoms, and co‐occurring condition(s). Associations between OSA severity and caregiver‐reported sleep symptoms and co‐occurring conditions were explored.

**Results:**

Of the 526 children (mean age = 5.69 years) with valid polysomnogram results, 419 (79.7%) were diagnosed with OSA based on the criteria of OAHI ≥ 2 events/h and 268 (51.0%) with moderate/severe OSA. Mean OAHI was 10.1 events/h (SD 14.1). Witnessed apnea was positively associated with moderate/severe OSA, whereas restless sleep was negatively associated. There is a significant positive association between higher body mass index (BMI) category and more severe OSA (*p* = 0.02). Conversely, there was a negative association between moderate/severe OSA and a history of feeding difficulties (*p* = 0.037).

**Conclusions:**

In this large cohort of children with DS, we confirm a high prevalence of OSA. Additionally, caregiver‐reported witnessed apneic events occurring most nights and BMI category severity were associated with worsened OSA severity.

## 1 Introduction

Down syndrome (DS) is the most frequently occurring chromosomal abnormality. Approximately 1 in 700 babies born in the United States each year are diagnosed with DS [1]. Children with DS can have many co‐occurring medical conditions as a result of altered gene expression of chromosome 21 [2]. These children often have anatomical and physiological features that predispose them to obstructive sleep apnea (OSA), including mid‐facial and mandibular hypoplasia, narrow nasopharyngeal airway, adenotonsillar hypertrophy, relative macroglossia, laryngomalacia, subglottic stenosis, tracheobronchomalacia, obesity, and generalized hypotonia [3, 4, 5]. A 2018 meta‐analysis included 18 studies and approximately 1200 children with DS and reported that the incidence of OSA in children with DS ranges from 69% to 76%, depending on whether an apnea hypopnea index (AHI) threshold of 1, 1.5, or 2 events/h is used [3]. In patients with OSA, 50% of cases are moderate or severe (AHI ≥ 5 events/h) [3].

If left untreated, OSA may lead to adverse outcomes in adulthood, including refractory hypertension, coronary artery disease, cerebrovascular accidents, arrhythmias, congestive heart failure, and pulmonary hypertension [6, 7]. Evidence of neurological impact can manifest as early as childhood: one study of typically developing children with snoring versus their non‐snoring peers found that children with higher AHI results performed worse on tests of differential ability scales, verbal and nonverbal performance, and global conceptual ability [8, 9]. Similarly, studies of children with DS have detected associations between OSA severity and impaired language, working memory, emotional control, and executive functioning [10, 11, 12].

To optimize outcomes of patients with DS, providers must be able to reliably diagnose OSA and effectively intervene. Unfortunately, caregiver reports of sleep symptoms in children with DS have been shown to have poor sensitivity [13, 14]. For instance, one group described that 69% of parents denied any sleep problems with their child; however, 54% of those children had abnormal polysomnograms (PSG) [15]. Providers are unable to discern based on history alone which patients are more or less likely to have OSA. The American Academy of Pediatrics (AAP) therefore recommends that all children with DS should undergo a screening PSG between the ages of 3 and 4 years [2].

In this study, we examine a large cohort of children with DS and evaluate the results of their first PSGs. These studies were performed at moderate altitude, which is defined as elevations between 1500 and 2500 m above sea level (masl) [16]. The primary aim of this study was to identify demographic characteristics, caregiver‐reported sleep symptoms, and co‐occurring conditions associated with moderate or severe OSA. We hypothesized that the presence of co‐occurring conditions and/or sleep‐related symptoms in children with DS would be associated with more severe OSA.

## 2 Methods

### 2.1 Participants

This single‐center, retrospective, descriptive study was approved by the Colorado Multiple Institutional Review Board, which determined an exempted requirement for informed consent (COMIRB #16‐2476). Patients were identified from a DS clinic (Sie Center for Down Syndrome) within a tertiary care hospital (Children's Hospital of Colorado [CHCO]). Electronic medical records of 1975 patients with DS were reviewed. Children met inclusion criteria if: (a) they had been seen for at least one appointment at the Sie Center for Down Syndrome; (b) they underwent their first diagnostic PSG at CHCO between 2012 and 2021; (c) they were between ages 1 and 15 years at the time of the PSG; (d) at minimum, data was available for obstructive apnea hypopnea index (OAHI), central apnea hypopnea index (CAHI), and total sleep time (TST); and (f) TST was ≥ 120 min. PSGs were performed and scored as previously described, aligned with the American Academy of Sleep Medicine (AASM) guidelines at the time of interpretation [17, 18]. All sleep studies were performed at a CHCO sleep laboratory, located at an altitude between 1642 and 2087 masl.

### 2.2 Data Collection

**Figure 1:**
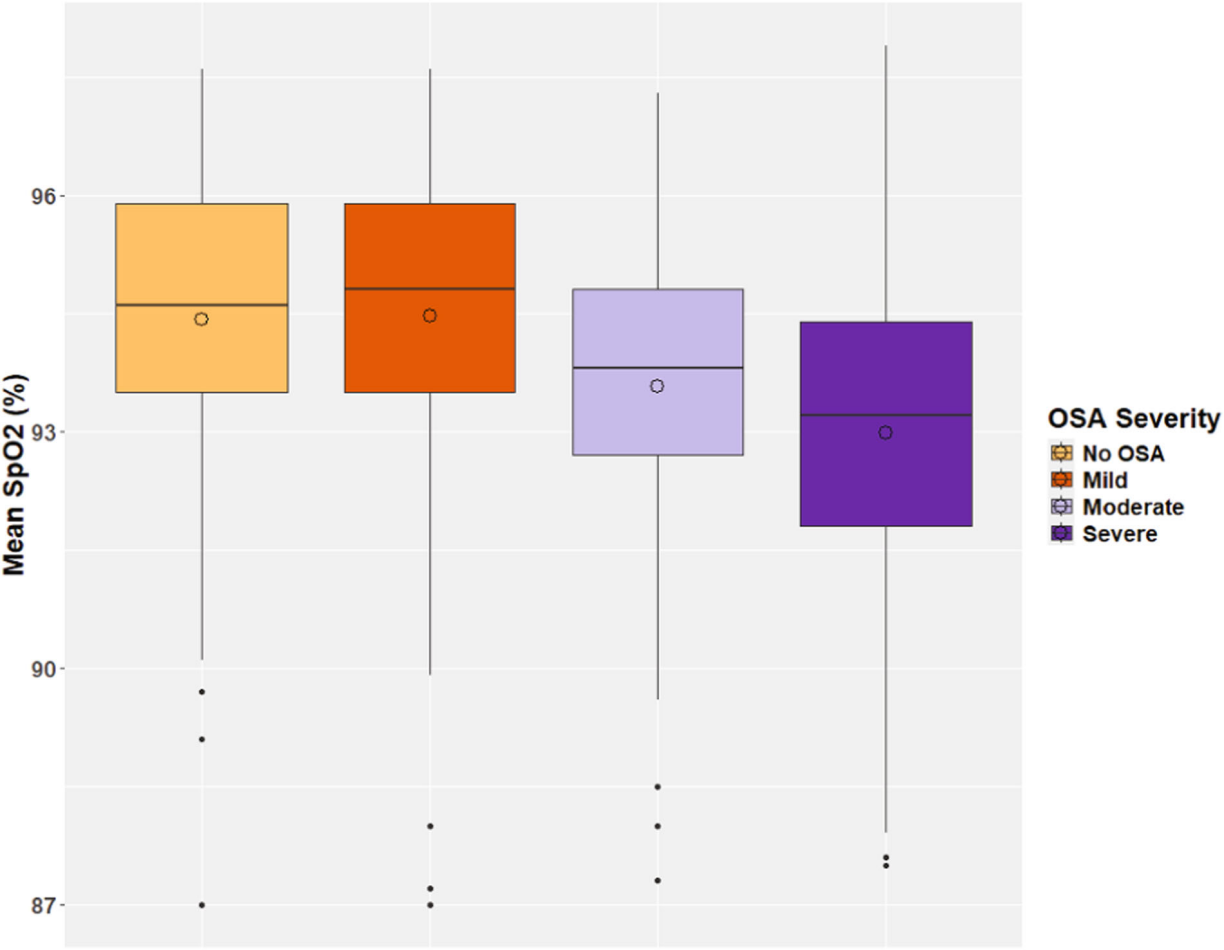
Boxplot of mean oxyhemoglobin saturation (SpO_2_) by OSA severity.

Information regarding past medical history and co‐occurring conditions was obtained via parent interviews during appointments at the Sie Center for Down Syndrome and/or by retrospective chart review. The following co‐occurring conditions were evaluated: congenital heart defects, gastrointestinal malformations, feeding problems, eustachian tube dysfunction, laryngomalacia, autoimmune disorders, thyroid disorders, atlantoaxial instability, seizure disorders, episodes of pneumonia, and episodes of pulmonary hypertension. Caregiver sleep symptom surveys had been previously completed on the night of the PSG (Supporting Information S1: Figure 1). Parents were asked to rate how frequently—ranging from never to always—their child exhibits the following sleep‐related symptoms: stops breathing during sleep, snores, restless sleep, sweating when sleeping, fatigue, morning headache, nocturnal enuresis, mouth‐breathing, foul breath, kicks legs in sleep, awakenings, frequent repositioning at night, trouble getting up in the morning, falls asleep in school, naps after school, and leg cramps/leg pain. Study data were collected and managed using Research Electronic Data Capture (REDCap) electronic data capture tools hosted at the University of Colorado [19]. REDCap is a secure, web‐based software platform designed to support data capture for research studies.

### 2.3 Data Analysis

Similar to the Childhood Adenotonsillectomy Trial (CHAT), OSA was defined as OAHI ≥ 2 events/h [20]. Hypoventilation was defined as end tidal carbon dioxide (ETCO_2_) > 50 mmHg for > 50% TST [17]. CSA was defined as central apnea index (CAI) ≥ 5 events/h [21]. Patients met criteria for periodic breathing (PB) if they exhibited PB for > 5% TST.

Descriptive statistics are reported as mean (SD) for numeric variables. Categorical variables are reported as frequency (%). Descriptive statistics are stratified by OAHI severity, defined as OAHI < 2 = none, 2 ≤ OAHI < 5 = mild, 5 ≤ OAHI < 10 = moderate, and OAHI ≥ 10 = severe. Body mass index (BMI) classes were defined as follows using the Centers for Disease Control (CDC) growth charts: underweight or healthy < 85th percentile‐for‐age; overweight ≥ 85 to < 95th percentile‐for‐age; and obese ≥ 95th percentile‐for‐age.

Chi‐squared, Fisher's exact, or two‐sample Student *t*‐tests were performed to determine if demographic variables (Table 1) or cardiopulmonary and sleep quality measures (Table 2) differed between OAHI severity categories (none/mild vs. moderate/severe). Nonparametric tests for numeric variables were not considered due to large sample sizes. For the association between BMI class and OAHI severity, a Mantel−Haenszel chi‐square test for the linear association was used. Lastly, analysis of variance (ANOVA) was performed to compare the mean SpO_2_ and percent time SpO_2_ below 90% between OSA severity categories. A *p*‐value of < 0.05 was considered statistically significant.

**Table 1:** Patient demographics and co‐occurring conditions.

	None/Mild (*N* = 258)	Moderate/Severe (*N* = 268)	*p* Value (*N* = 0)	Overall (*N* = 526)
Age (years)				
Mean (SD)	5.51 (3.47)	5.86 (4.04)	0.288	5.69 (3.77)
Sex				
Female	128 (49.6%)	116 (43.3%)	0.171	244 (46.4%)
Male	130 (50.4%)	152 (56.7%)		282 (53.6%)
Race				
White	183 (71.8%)	178 (68.2%)	0.444	361 (70.0%)
Black or African American	7 (2.7%)	13 (5.0%)		20 (3.9%)
Asian	3 (1.2%)	6 (2.3%)		9 (1.7%)
Other	62 (24.3%)	64 (24.5%)		126 (24.4%)
Ethnicity				
Hispanic or Latino	107 (42.0%)	99 (38.1%)	0.418	206 (40.0%)
Not Hispanic or Latino	148 (58.0%)	161 (61.9%)		309 (60.0%)
Primary language				
English	194 (76.4%)	206 (78.3%)	0.908	400 (77.4%)
Spanish	56 (22.0%)	53 (20.2%)		109 (21.1%)
Other	4 (1.6%)	4 (1.5%)		8 (1.5%)
BMI class^a^, ^b^				
Normal	154 (60.2%)	139 (51.9%)	0.0221	293 (55.9%)
Overweight	55 (21.5%)	58 (21.6%)		113 (21.6%)
Obese	47 (18.4%)	71 (26.5%)		118 (22.5%)
History of heart defects				
No	19 (7.6%)	23 (9.1%)	0.638	42 (8.4%)
Yes	232 (92.4%)	229 (90.9%)		461 (91.7%)
Type of heart defect				
AV canal or VSD	95 (40.9%)	92 (40.2%)	0.941	187 (40.6%)
Other	137 (59.1%)	137 (59.8%)		274 (59.4%)
History of pulmonary hypertension				
No	103 (60.2%)	114 (65.5%)	0.366	217 (62.9%)
Yes	68 (39.8%)	60 (34.5%)		128 (37.1%)
History of feeding problems				
No	78 (39.6%)	91 (46.2%)	0.222	169 (42.9%)
Yes	119 (60.4%)	106 (53.8%)		225 (57.1%)
History of GI malformations				
No	163 (78.0%)	151 (72.9%)	0.279	314 (75.5%)
Yes	46 (22.0%)	56 (27.1%)		102 (24.5%)
History of thyroid disorder				
No	117 (57.4%)	109 (53.2%)	0.453	226 (55.3%)
Yes	87 (42.6%)	96 (46.8%)		183 (44.7%)
History of seizure disorder				
No	167 (91.8%)	153 (90.0%)	0.698	320 (90.9%)
Yes	15 (8.2%)	17 (10.0%)		32 (9.1%)

^a^ BMI class was compared using a Mantel−Haenszel chi‐square.

^b^ BMI classes were defined as follows using CDC growth charts: Underweight or healthy < 85th percentile‐for‐age; overweight ≥ 85 to < 95th percentile‐for‐age; and obese ≥ 95th percentile‐for‐age.

**Table 2:** Cardiopulmonary and sleep quality measures from polysomnography.

	None/Mild (*N* = 258)	Moderate/Severe (*N* = 268)	*p* Value	Overall (*N* = 526)
AHI (events/h)	3.53 (2.16)	18.8 (16.7)	< 0.001	11.3 (14.2)
OAHI (events/h)	2.37 (1.38)	17.6 (16.5)	< 0.001	10.1 (14.1)
CAHI (events/h)	1.18 (1.59)	1.25 (1.82)	0.662	1.22 (1.71)
SpO_2_, mean	94.5 (1.9)	93.2 (2.0)	< 0.001	93.8 (2.0)
SpO_2_, minimum	83.5 (5.24)	78.3 (7.64)	< 0.001	80.8 (7.08)
Percent of time SpO_2_ < 90%	5.29 (15.0)	12.2 (18.7)	< 0.001	8.83 (17.3)
End‐tidal CO_2_, max	49.4 (5.6)	50.3 (4.9)	0.0487	49.9 (5.3)
End‐tidal CO_2_, mean	41.2 (5.0)	41.8 (6.2)	0.199	41.5 (5.7)
End tidal CO_2_, percent TST > 50	0.3 (0, 5.0)	0.1 (0, 2.9)	0.163	0.2 (0, 3.4)
Periodic breathing: %TST	0.1 (0, 0.8)	0 (0, 0.68)	0.297	0.1 (0, 0.7)
Heart rate	86.6 (13.8)	90.2 (13.9)	0.003	88.4 (13.9)
Total sleep time (TST)	409.0 (75.6)	351.0 (85.6)	< 0.001	379.0 (85.8)
Sleep efficiency (%)	83.3 (9.7)	79.9 (11.2)	< 0.001	81.6 (10.6)
Sleep latency (mins)	25.9 (34.9)	28.9 (46.1)	0.405	27.4 (41.0)
REM latency (mins)	140 (73.8)	147 (66.4)	0.227	144 (70.2)

*Note:* Reported as median (Q1, Q3) or mean (SD).

Multivariable logistic regressions were fit to identify associations between sleep symptoms or co‐occurring diagnoses and OAHI severity, which was classified as none/mild versus moderate/severe. For all multivariable models, age, sex, previous ENT surgery (T&A, tonsillectomy, adenoidectomy, tongue base reduction, lingual tonsillectomy, or supraglottoplasty), BMI class, and whether the patient lives at an altitude > 1828 masl were considered for covariates. Backward selection was used to iteratively remove insignificant predictors until identifying a model retaining only predictors with a *p* < 0.05. Only results from the multivariable models with a significant effect of sleep symptoms or co‐occurring diagnoses following backward selection are reported. All categorical sleep symptom predictors were compressed into never/not often (0 nights/week), sometimes (1−2 nights/week), and often/always (≥ 3 nights/week). For variables that had a “Not sure” or “Not assessed” options, these values were treated as missing, and patients who selected these options were excluded from the associated regression. *p* values from regressions were not adjusted to account for multiple testing.

## 3 Results

Of the 1975 children enrolled in the Sie Center for Down Syndrome clinic database, 526 patients met our inclusion criteria. Of the patients who were excluded, the most common reasons were: did not have a PSG at CHCO (*n* = 1039), timing of first PSG was before 2012 (*n* = 291), age outside the range (*n* = 251), and TST < 120 min (*n* = 68). In those included, the mean age at the time of first PSG was 5.69 years. Most patients (70.0%) identified their race as White, followed by other (24.4%), Black or African American (3.9%), and Asian (1.7%). Roughly 40% of patients identified as Hispanic/Latino. English was listed as the primary language by 77.4% of families. When comparing patients with none or mild OSA to those with moderate or severe OSA, the groups did not differ significantly with respect to age, sex, race, ethnicity, or primary language (Table 1). However, there was a statistically significant association between BMI class and OAHI severity. Those with no or mild OSA had higher rates of normal BMI class, and those with moderate or severe OSA had higher rates of obesity (*p* = 0.02).

Thirty‐one percent (*n* = 164) of the children included in the study underwent an upper airway surgery (i.e., T&A, adenoidectomy, tonsillectomy, tongue base reduction, lingual tonsillectomy, or supraglottoplasty) before their first PSG. The cardiopulmonary variables were contrasted between patients with a history of prior airway surgery versus those without. No significant difference was detected between the groups' measurements for AHI, OAHI, CAI, PB, or percent time with oxygen saturation (SpO_2_) < 90% (Supporting Information S1: Table 1). The history of prior surgery was considered as a covariate in all regression models to account for potential confounding.

Overall, 419 (79.7%) of patients met diagnostic criteria for OSA, including 164 children who had previously undergone upper airway surgery. OAHI cut‐offs were used to characterize patients as having mild, moderate, or severe OSA. In our sample, 268 patients had moderate or severe OSA, with the complete breakdown of 151 (28.7%) mild, 105 (20.0%) moderate, and 163 (31.0%) severe. The mean OAHI was 10.1 events/h (Table 2). The mean SpO_2_ while asleep was approximately 94%, and patients spent an average of 8.8% TST with oxygen saturation levels < 90%. Twenty patients (4.0%) met diagnostic criteria for clinically significant CSA, and of these patients, 11 also had OSA [21]. PB was diagnosed in 23 (5.0%) of children. Hypoventilation was confirmed in 46 (9.3%) of patients; 20 of whom also had moderate/severe OSA.

**Figure 2:**
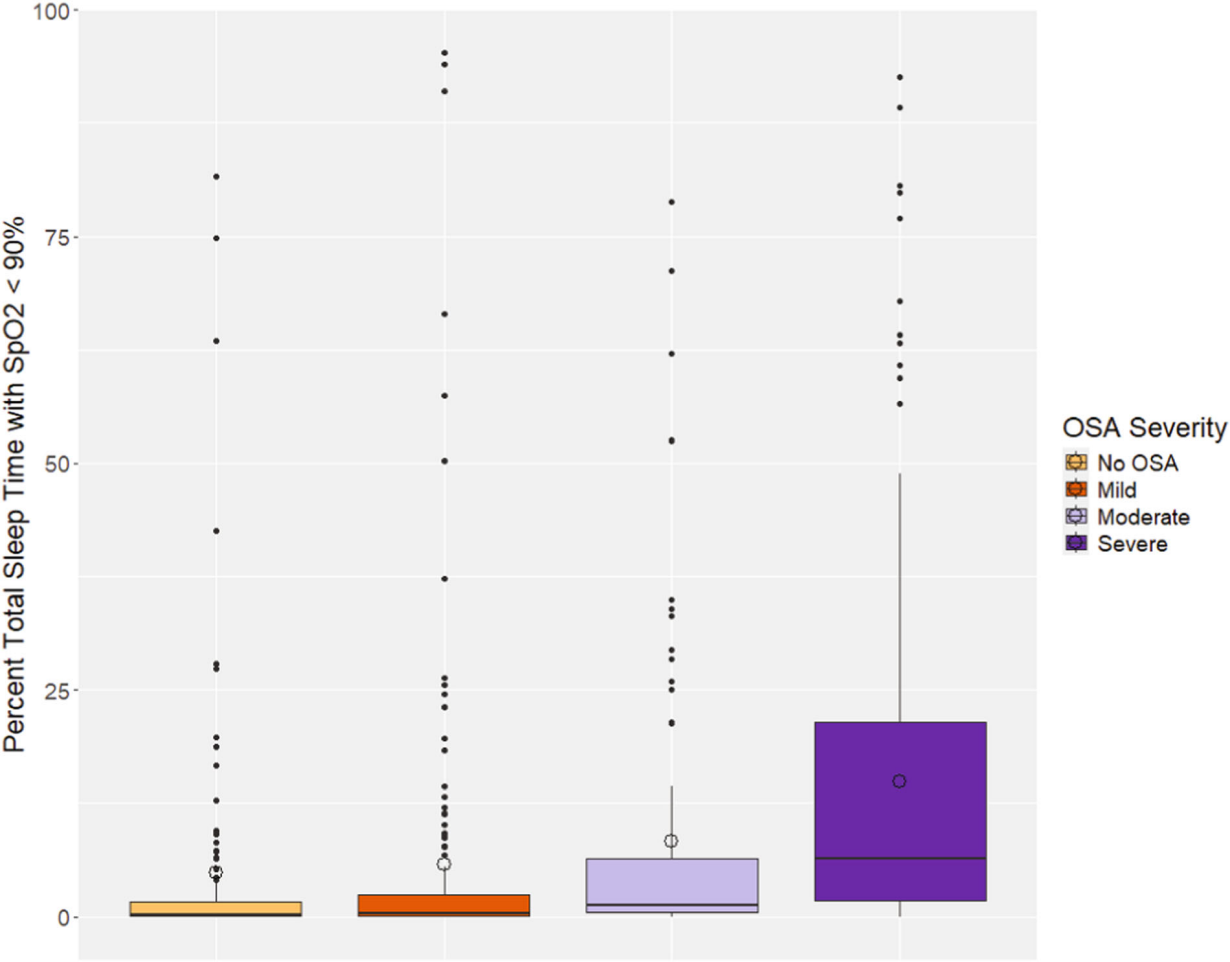
Boxplot of percent total sleep time with SpO_2_ < 90% by OSA severity.

When comparing cardiopulmonary and sleep quality variables by OSA severity (none/mild group vs. moderate/severe group), significant differences were detected between the patients' AHI, OAHI, mean SpO_2_, minimum SpO_2_, percent time with SpO_2_ < 90%, maximum ETCO_2_, average heart rate, TST, and sleep efficiency (Table 2, all *p* < 0.05). For children without OSA or with only mild OSA, their average oxygen saturations were essentially equal (94.8% and 94.6% respectively). However, the average SpO_2_ did decline progressively for those with moderate and severe OSA (Figure 1). The percentage of sleep time with SpO_2_ < 90% increased as OSA severity worsened (Figure 2). Additionally, ANOVA revealed a statistically significant difference (both *p* < 0.001) when comparing both mean SpO_2_ and percent time with SpO_2_ < 90% between OSA severity groups (none, mild, moderate, and severe).

Logistic regressions assessed associations between sleep symptoms and OSA severity. Caregiver reports of “stops breathing” and “restless sleep” were found to be significantly associated (*p *< 0.05) with OSA severity, while adjusting for relevant covariates. Specifically, the odds of having moderate or severe OSA are greater by a factor of 2.39 (95% CI: 1.16, 4.92; *p* = 0.018) for patients reporting often or always stopping breathing compared to those that never or not often observe this behavior (Supporting Information S1: Table 2). The odds of moderate or severe OSA are lower by a factor of 0.52 (95% CI: 0.29, 0.92; *p* = 0.027) for patients reporting restless sleep sometimes compared to those reporting this never or not often, while adjusting for BMI class (Supporting Information S1: Table 3).

**Figure 3:**
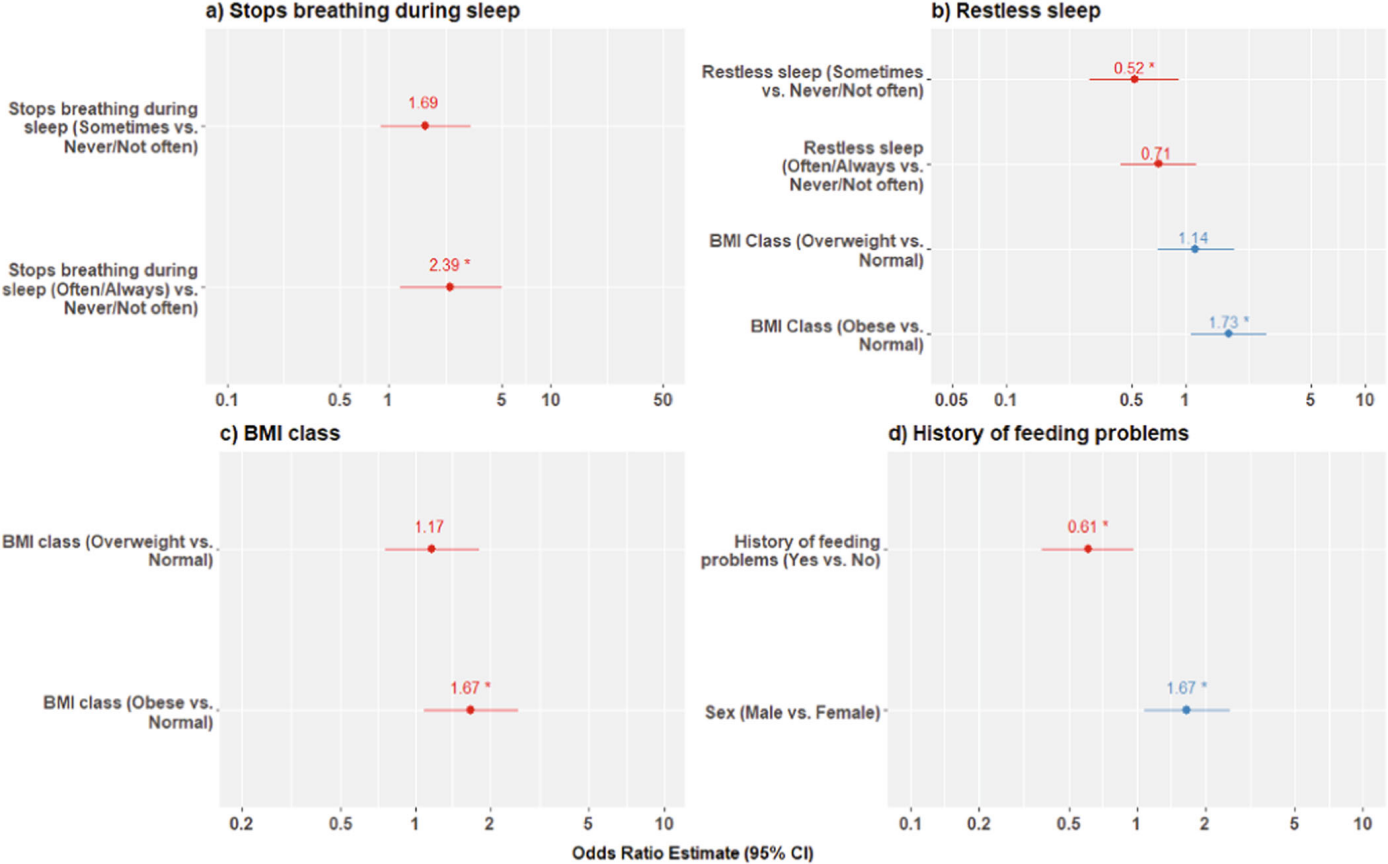
Forest plots of OR estimates and 95% CIs from logistic regressions to predict moderate/severe OAHI. Odds ratios are relative to “Never/Not often (< 1 night/week)” and “No” reference groups for sleep symptoms and history of feeding problems, respectively.

The same process was repeated for co‐occurring diagnoses (Supporting Information S1: Table 3). Logistic regression analyses identified significant associations between the following co‐occurring diagnoses and moderate/severe OSA, while adjusting for relevant covariates: BMI class and history of feeding problems. Patients who were obese were at significantly greater odds of moderate or severe OSA by a factor of 1.67 (95% CI: 1.09, 2.59; *p* = 0.02) compared to those with a normal BMI (Supporting Information S1: Table 4). There were no significant differences in odds of moderate or severe OSA for patients that were overweight compared to normal weight (OR: 1.17; 95% CI: 0.76, 1.81; *p* = 0.48). Patients with a history of feeding problems were at significantly lower odds of having moderate or severe OSA by a factor of 0.61, while adjusting for sex (95% CI: 0.38, 0.97; *p* = 0.037; Supporting Information S1: Table 5). Figure 3 shows forest plots of the odds ratios and 95% CI for the models with a *p* < 0.05 for caregiver‐reported sleep symptom or co‐occurring condition.

## 4 Discussion

Our results confirmed a high prevalence of OSA in children with DS. Of the 526 patients included in our study, 79.7/% had OSA with an OAHI ≥ 2 events/h). This prevalence is slightly higher than the prevalence range of 69%−76% that Lee et al previously reported in a 2018 meta‐analysis [3]. However, when using a lower OAHI cut‐off of ≥ 1 event/h, 90.3% of our patients were diagnosed with OSA.

OAHI values may increase due to a moderately high altitude. The compensatory hyperventilation associated with living at a higher altitude may predispose children to conditions of sleep‐disordered breathing (SDB), such as OSA, CSA, and PB. Burg et al. examined the respiratory and PSG data of 53 healthy, non‐snoring children ages 3−5 years who had lived near Denver, Colorado, for longer than 1 year. Compared to a group of children living at sea level, the moderate altitude‐dwelling children demonstrated minor changes in respiratory patterns during sleep, higher average CO_2_ levels, more central respiratory events, increased PB, and more frequent oxygen desaturation [22]. Similarly, Hughes et al. performed a crossover study of 10 children with SDB that compared results from an in‐lab PSG (median 1644 masl) to a significantly higher elevation at‐home PSG (median 2531 masl). The median AHI increased from 2.40 events/h during the in‐lab PSG to 10.95 events/h during the home studies. In general, measures of OAHI, CAHI, oxygenation, sleep fragmentation, and pulse rate were worse on the home studies [18]. These findings suggest that even an altitude change of less than 1000 masl can alter respiratory physiology during sleep.

Similar changes have been noted in populations living at even higher altitudes. A systematic review examined the findings of six studies that evaluated the physiology in sleep of children living at high altitude (> 2500 masl) in the Andes, South America. Both the CAI and PB were noted to increase during infancy but then decrease with age. Additionally, the frequency of PB increased with rising altitude [23]. One study specifically studied SDB in children with DS living at high altitudes by evaluating 53 children with DS who were referred to a high‐altitude sleep lab located at 2640 masl in Colombia. Based upon an OAHI threshold of ≥ 2 events/h, over 90% of patients were diagnosed with OSA, and 67.9% of cases qualified as moderate or severe [24]. Interestingly, the degree of OSA in our cohort appears higher than similar populations of children with DS living at sea level, but lower than populations living at high altitude. This finding supports that altitude has a dose‐dependent effect on respiratory physiology.

In our cohort, 20 children (3.95%) met criteria for CSA, and 11 (55%) of those patients also had moderate/severe OSA. A similar portion of children (*n* = 23 or 5.0%) were diagnosed with PB. Interestingly, our results for CSA fall within the range of the reported prevalence in the general pediatric population (1%−5%) [23]. This finding was counterintuitive to our hypothesis and differed from the results of other studies. For instance, Posada et al. reported that CSA was present in 11.3% of their patients with DS at high altitude, and PB was present in 15% [24]. The difference in altitude between our center and Colombia may explain this. However, Hizal et al. also reported a higher incidence of CSA (12.3%) in their cohort, and their facility is located at only 850 masl [25]. Another possible explanation could be that both studies included infants younger than 12 months of age, whereas our study purposefully excluded this age group.

Analysis of gas exchange revealed that both oxygenation measurements (mean SpO_2_ and percent time with SpO_2_ < 90%) decreased with higher OAHI results. ANOVA testing confirmed that these variables differed significantly between the OSA severity groups (none, mild, moderate, or severe). However, the difference between none and mild OSA does not appear to be clinically significant. As shown in Figure 1, the mean SpO_2_ for these two groups are essentially equal (94.8% vs. 94.6%). These findings likely support previously demonstrated limitations of using pulse oximetry to screen for OSA [26, 27]. A separate, predictive study and analysis would be necessary to say with certainty.

Our study aimed to identify variables associated with moderate or severe OSA that might inspire easier methods to diagnose OSA in children with DS. Patient demographics, parental‐reported sleep symptoms, and co‐occurring conditions were all examined. The variables of age, sex, race, ethnicity, and primary language spoken at home did not differ significantly between patients with none/mild OSA versus moderate/severe OSA. A significant association between BMI class (underweight/healthy, overweight, and obese) and OSA severity (none/mild vs. moderate/severe) was detected. Many prior studies have analyzed the relationship between BMI and OSA severity. Chamseddin et al. evaluated 106 children with DS between the ages of 2−18 years and found that children with obesity were twice as likely to have severe OSA [28]. Maris et al. detected a significant association between BMI *z*‐score and initial OAHI [29]. Skoto et al. used a logic‐learning machine to generate a model to predict which patients with DS were unlikely to have moderate or severe OSA, and therefore, were less likely to benefit from undergoing a PSG. The variables of height, weight, and BMI were all included in the final model [30]. Hsieh et al. reported a trend in 34 patients with DS that those with OSA had a higher weight/BMI‐for‐age [31].

Lastly, sleep symptom surveys, completed the night of the PSG by a parent or guardian, were analyzed to determine if the presence of sleep‐related symptoms is associated with moderate/severe OSA. Children who were reported to stop breathing during sleep often/always (3−7 nights/week) were at 2.4 (95% CI: 1.18, 5.03) times greater odds of having moderate or severe OSA, compared to children whose parents say they never or not often (< 1 night/week) stop breathing during sleep. Children whose parents noted a restless sleep trended toward being less likely to have moderate/severe OSA. This association was only significant for children who sometimes (1−2 nights/week) have restless sleep, but not for those with restless sleep often/always (3−7 nights/week). We suspect that this negative trend between OSA severity and restless sleep exists because restless sleep is nonspecific to OSA. Alternatively, parents may have interpreted the question of “restless sleep” differently [32, 33]. In the future, we hope to compare subjective reports of restlessness with objective PSG measurements to better contextualize restless sleep in the DS population. Of children whose parents denied any sleep‐related symptoms at all, 45.5% were diagnosed with moderate or severe OSA. Altogether, these results confirm what has been shown by many prior studies: that parental‐reported sleep symptoms in children with DS are poorly sensitive for moderate/severe OSA.

While the AAP's recommendations are critical to support the diagnosis of OSA for many children with DS, they are not without limitations [2]. These guidelines fail to address how often screening sleep studies should be performed in patients whose first PSG as a young child was negative for OSA. Patients with a normal PSG may not undergo another sleep study despite the presence of symptoms [31]. Even patients who benefit from surgical intervention will require repeat evaluations: Dialla et al. reported that at least 30% of children with no to mild OSA (OAHI < 5 events/h) following a tonsillectomy will progress to moderate or severe OSA (OAHI ≥ 5 events/h) within 2 years [34]. Additionally, studies are often difficult to access for patients and families due to cost [31], time commitment, travel, and limited pediatric sleep laboratories. Patients with DS, specifically, may be unable to tolerate a PSG due to sensory processing difficulties. For these reasons, there is a need for approaches that use family‐reported information to help identify children who are likely experiencing moderate/severe OSA and would benefit from completing a PSG to guide treatment. Several studies have attempted to identify symptoms or risk factors associated with OSA, but the results have been largely conflicting or difficult to apply clinically [25, 30].

There were limitations to our study that are inherent to any retrospective review. We were not able to control for parameters that may have influenced the health measure results. Though there is the possibility of inconsistencies in the historical medical records and testing evaluation reports, data were reviewed and verified by trained members of the Sie Center research team. Additionally, nearly one‐third of our patients had undergone upper airway surgery (T&A, adenoidectomy, tonsillectomy, tongue base reduction, lingual tonsillectomy, and/or supraglottoplasty) before their first PSG. When comparing sleep study variables between the group with prior upper airway surgery versus the group without, there was not a significant difference in AHI, CAHI, OAHI, PB, or percent time with oxygen saturation less than 90%. To account for potential confounding, the history of surgery was considered as a covariate in all regression models. Without data on sleep architecture or staging, we were unable to investigate the impact of REM stage sleep. Lastly, the sleep intake form utilized by our sleep labs for the last 20 years has not been validated (Supporting Information S1: Figure 1).

In conclusion, our findings confirm a high prevalence of OSA in children with DS with a high rate of moderate and severe OSA. The major strength of our study is its strict inclusion criteria and large sample size (*n* = 526). Additionally, our results demonstrate an association with obesity and OSA in this cohort, highlighting the importance of discussing anticipatory guidance regarding weight gain with caregivers. Lastly, caregiver perception of “stops breathing” was associated with more severe OSA, while generally restless sleep was not. Further work is needed to improve both quality of life and quality of sleep for children with DS and their families. This study includes validating patient symptom scales and other biomarkers of OSA, as well as improving tolerability of PSGs and OSA treatment.

## Author Contributions

**Taylor A. Curry:** investigation, writing – original draft, methodology, writing – review and editing, data curation, conceptualization. **Emily Cooper:** methodology, writing – review and editing, formal analysis, data curation. **John Brinton:** methodology, formal analysis. **Kristine Wolter‐Warmerdam:** conceptualization, data curation, writing – review and editing, project administration. **Norman R. Friedman:** methodology, writing – review and editing, conceptualization, supervision. **Stephen M. M. Hawkins:** writing – review and editing, methodology, conceptualization, supervision. **Francis Hickey:** conceptualization, writing – review and editing, supervision, project administration. **Benjamin H. Hughes:** writing – review and editing, methodology, conceptualization, supervision. **Emily M. DeBoer:** conceptualization, investigation, writing – review and editing, methodology, supervision.

## Conflicts of Interest

Dr. Emily M. DeBoer is a consultant and founder of EvoEndoscopy and has patents related to unsedated endoscopy. Dr. Emily M. DeBoer is a consultant for Boeringer Ingelheim and Parexel, not related to this project. The other authors declare no conflicts of interest.

## Supporting information


**Supplemental Figure 1:** Sleep Intake Parental Questionnaire. **Supplemental Table 1:** Comparison of PSG measures for patients with a prior ENT surgery (T&A, tonsillectomy, adenoidectomy, tongue base reduction, lingual tonsillectomy, or supraglottoplasty). **Supplemental Table 2:** Logistics regression results to predict moderate/severe OAHI based on frequency of stopping breathing. **Supplemental Table 3:** Logistics regression results to predict moderate/severe OAHI based on frequency of restless sleep. **Supplemental Table 4:** Logistics regression results to predict moderate/severe OAHI based on BMI class. **Supplemental Table 5:** Logistics regression results to predict moderate/severe OAHI based on history of feeding problems.

## Data Availability

The data that support the findings of this study are available from the corresponding author upon reasonable request.
