# Supplementary material for: Co‐Occurring Conditions and Sleep Symptoms Associated With Obstructive Sleep Apnea in Children With Down Syndrome
Source: Pediatr Pulmonol. 2025 Nov 24;60(11):e71376. doi: 10.1002/ppul.71376 (PMC12645186; doi:10.1002/ppul.71376)
Supplement: Supplementary file 1 — Supplemental Figure 1: Sleep Intake Parental Questionnaire. Supplemental Table 1: Comparison of PSG measures for patients with a prior ENT surgery (T&A, tonsillectomy, adenoidectomy, tongue base reduction, lingual tonsillectomy, or supraglottoplasty). Supplemental Table 2: Logistics regression results to predict moderate/severe OAHI based on frequency of stopping breathing. Supplemental Table 3: Logistics regression results to predict moderate/severe OAHI based on frequency of restless sleep. Supplemental Table 4: Logistics regression results to predict moderate/severe OAHI based on BMI class. Supplemental Table 5: Logistics regression results to predict moderate/severe OAHI based on history of feeding problems. [file PPUL-60-0-s001.doc]

**Supplemental Figure 1: Sleep Intake Parental Questionnaire**

**
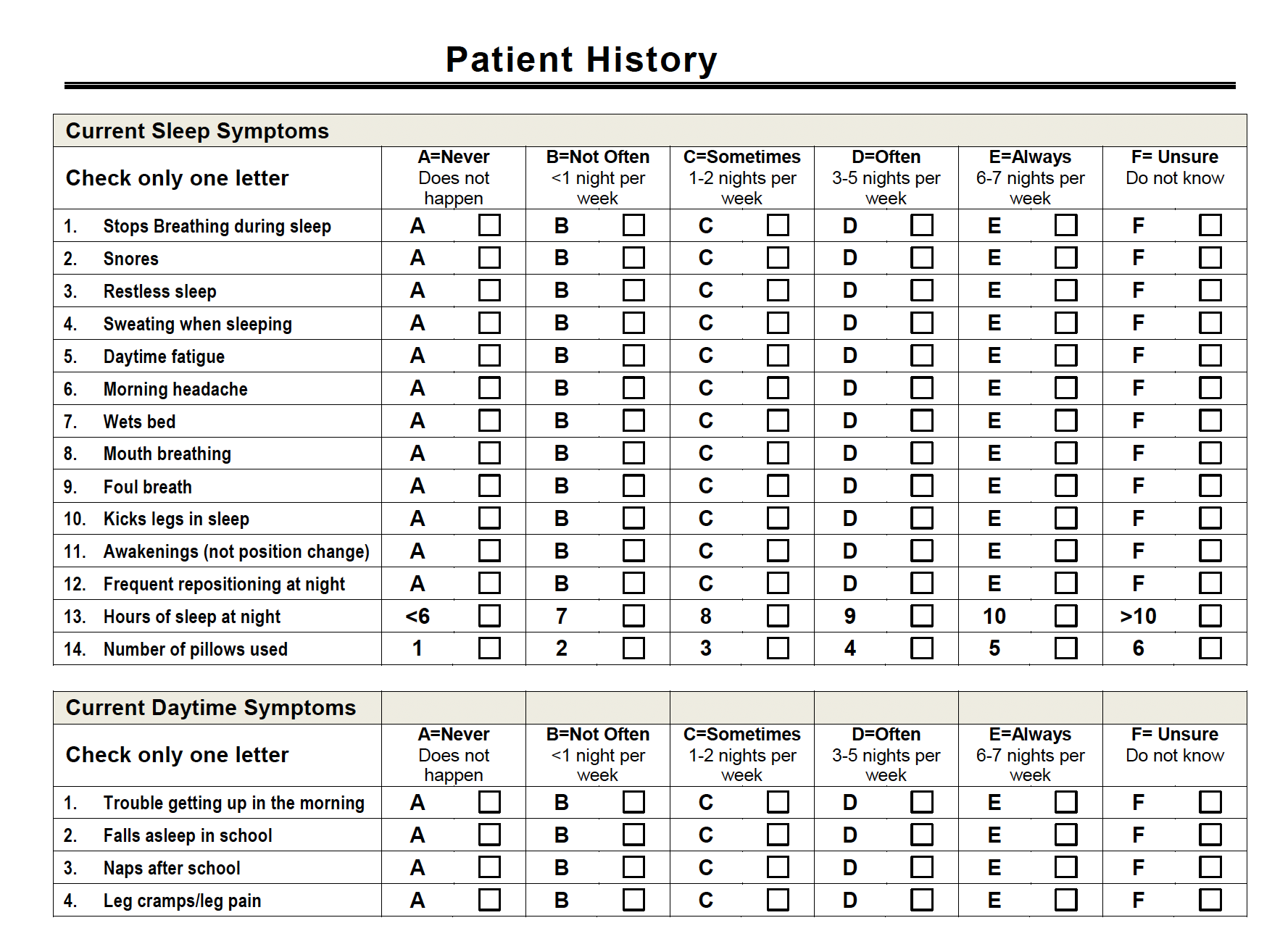
**

**Supplemental Table 1: Comparison of PSG measures for patients with a prior ENT surgery (T&A, tonsillectomy, adenoidectomy, tongue base reduction, lingual tonsillectomy, or supraglottoplasty)**

|  | **Prior ENT surgery (N=164)** | **None (N=362)** | **P-value (N=0)** |
| --- | --- | --- | --- |
| **OAHI (events/hr)** |  |  |  |
| **Mean (SD)** | **10.1 (12.7)** | **10.2 (14.7)** | **0.943** |
| **Median (Q1, Q3)** | **4.95 (2.70, 12.0)** | **5.10 (2.20, 12.5)** |  |
| **CAHI (events/hr)** |  |  |  |
| **Mean (SD)** | **1.11 (1.72)** | **1.26 (1.70)** | **0.361** |
| **Median (Q1, Q3)** | **0.500 (0.100, 1.48)** | **0.700 (0.200, 1.63)** |  |
| **AHI (events/hr)** |  |  |  |
| **Mean (SD)** | **11.2 (12.8)** | **11.4 (14.9)** | **0.872** |
| **Median (Q1, Q3)** | **6.50 (3.60, 14.1)** | **6.50 (3.40, 13.2)** |  |
| **Periodic breathing (% time)** |  |  |  |
| **Mean (SD)** | **1.25 (4.59)** | **0.920 (2.68)** | **0.415** |
| **Median (Q1, Q3)** | **0.100 (0, 0.800)** | **0.100 (0, 0.700)** |  |
| **% Total sleep time with SpO2 < 90%** |  |  |  |
| **Mean (SD)** | **8.52 (17.3)** | **8.98 (17.3)** | **0.779** |
| **Median (Q1, Q3)** | **0.900 (0.200, 6.45)** | **1.25 (0.200, 8.85)** |  |

**Supplemental Table 2: Logistics regression results to predict moderate/severe OAHI based on frequency of stopping breathing**

| ***Predictors*** | ***Odds Ratios*** | ***95% CI*** | ***p-value*** |
| --- | --- | --- | --- |
| **Stops breathing** (vs. Never/Not often)  Sometimes, 1-2 nights per week | 1.69 | 0.90 – 3.22 | 0.107 |
| Often/Always, 3-7 nights per week | 2.39 | 1.18 – 5.03 | 0.018 |

**Supplemental Table 3: Logistics regression results to predict moderate/severe OAHI based on frequency of restless sleep**

| ***Predictors*** | ***Odds Ratios*** | ***95% CI*** | ***p-value*** |
| --- | --- | --- | --- |
| **Restless sleep** (vs. Never/Not often)  Sometimes, 1-2 nights per week | 0.52 | 0.29 – 0.92 | 0.027 |
| Often/Always, 3-7 nights per week | 0.71 | 0.44 – 1.15 | 0.167 |
| **BMI class** (vs. Normal weight)  Overweight | 1.14 | 0.70 – 1.86 | 0.603 |
| Obese | 1.73 | 1.08 – 2.82 | 0.025 |

**Supplemental Table 4: Logistics regression results to predict moderate/severe OAHI based on BMI class**

| ***Predictors*** | ***Odds Ratios*** | ***95% CI*** | ***p-value*** |
| --- | --- | --- | --- |
| **BMI class** (vs. Normal weight)  Overweight | 1.17 | 0.76 – 1.81 | 0.483 |
| Obese | 1.67 | 1.09 – 2.59 | 0.020 |

**Supplemental Table 5: Logistics regression results to predict moderate/severe OAHI based on history of feeding problems**

| ***Predictors*** | ***Odds Ratios*** | ***95% CI*** | ***p-value*** |
| --- | --- | --- | --- |
| **History of feeding problems** (vs. No)  Yes | 0.61 | 0.38 – 0.97 | 0.037 |
| **Sex** (vs. Female)  Male | 1.67 | 1.08 – 2.59 | 0.022 |
